# A Thematic Analysis of Ethical Issues Faced by Nursing Students in Clinical Practice

**DOI:** 10.1177/23779608261436765

**Published:** 2026-06-25

**Authors:** Elena Panayiotou, Maria Dimitriadou, Andreas Charalambous, Theologia Tsitsi

**Affiliations:** 1 Nursing Department, 447662 Cyprus University of Technology, Limassol, Cyprus

**Keywords:** ethics, nursing, nursing students, ethical issues, clinical practice

## Abstract

**Introduction:**

Ethical and moral complexities are common in clinical environments and shape nursing students’ professional development. Ethics education supports the development of critical thinking and moral reasoning in nursing students, enabling them to recognize and effectively address ethical issues in their professional practice. Studies have highlighted ethical issues nursing students face during their clinical placements.

**Objectives:**

This study aimed to explore the ethical issues nursing students faced during their four-year clinical training.

**Methods:**

A qualitative study was conducted using deductive thematic analysis, following Braun and Clarke's framework. A purposive sample of fourth-year undergraduate nursing students at a University in Cyprus (academic year 2022–2023). Data derived from written responses to an open-ended question embedded in the Nursing Ethics and Professional Legislation module.

**Results:**

With a response rate of 96.49% (55 students), analysis identified three themes: (1) Violation of Patients’ Rights (autonomy, informed consent, equality/religious freedom, access to medical records, and privacy), (2) Moral Distress (anxiety/pressure, awareness of the ideal but constrained action), and (3) Professional Liability and Nursing Codes of Ethics (communication failures, neglect, and lapses in duty of care).

**Conclusions:**

Nursing students observed violations of patient autonomy, informed consent, privacy, and equality, and experienced moral distress. Ethics education should emphasize core values, strengthen ethical decision making, and foster moral courage. In addition, academic and clinical settings need to provide emotional and professional support, to help students manage distress, and develop resilience as future professionals.

## Introduction

Ensuring patients’ fundamental rights is a key aspect of nursing education, leading to the development of ethical training strategies for nursing students (Martins et al., 2020). Ethics education helps nursing students develop critical thinking and moral reasoning, equipping them to identify and solve ethical problems in their practice (Cevik Durmaz, 2022). The primary objective of this training is to equip student nurses with the skills and knowledge necessary to address the ethical challenges they will experience in their profession, thereby enhancing patient care. The clinical learning environment is fundamental to the development of nursing students’ professional skills and ethical competence. As this setting often presents real-life ethical dilemmas, it is crucial to integrate ethics education into clinical training. Evidence suggests that ethical competence cannot be developed through theoretical instructions alone, instead, it requires engagement in experiential, practice-based learning (Hansen et al., 2024; Rasesemola & Molabe, 2025).

According to the National Cyprus Code of Nursing Ethics (NCCNE), which follows the International Council of Nurses (ICN) Code of Ethics (Stievano & Tschudin, 2019), nurses must adhere to six fundamental core principles: autonomy, nonmaleficence, utilitarianism, justice, loyalty, and confidentiality of sensitive information. Educators must ensure that students understand these ethical concepts and encourage them to reflect on ethical dilemmas critically, as they may encounter during clinical practice (Ramos et al., 2015).

Clinical training is an essential part of nursing education, providing students with the opportunity to develop professional competency. Well-structured and organized clinical placements are essential for providing students with the necessary support and learning experiences to develop the knowledge, skills, and attitudes required for their future careers (Immonen et al., 2019).

Nursing students develop key professional values during their studies, including ethics, responsibility, and expertise ([Bibr bibr13-23779608261436765][Bibr bibr13-23779608261436765]; Hansen et al., 2024). It is important to emphasize that Bioethics courses are essential for students as they prepare them to critically evaluate ethical challenges in healthcare, and provide them with the tools they need to make informed decisions (Ramos et al., 2015). Ethical codes, such as those created by Beauchamp and Childress (2013), emphasize patient autonomy, beneficence, avoiding harm, and ensuring fairness (Cheraghi et al., 2023). Nurses are also expected to maintain patient confidentiality, obtain informed consent, and advocate for their patients’ best interests.

Within the framework of the course, nursing students are educated on the concept of moral distress in the nursing profession, with emphasis on its manifestations and its impact on nurses across various clinical specialties. The concept of moral distress originates from the critical examination of the daily experiences of nursing professionals. According to Corley (2002), moral distress arises in nurses when they are aware of the ethically appropriate course of action for a patient but are unable to carry it out due to specific constraints. Corley (2002) found that moral distress can be detrimental, as it may lead nurses to consider leaving the profession. However, it can also have a positive impact by increasing nurses’ awareness of the ethical challenges they encounter in their daily clinical practice. Nursing students, like practicing nurses, experience moral distress when they face behaviors or situations in clinical settings that contradict the values, principles, and standards taught during their academic training (Heng & Shorey, 2023).

Bioethics education is essential for preparing nursing students to become ethical practitioners (Martins et al., 2020). It promotes critical thinking and decision-making skills, which are crucial when navigating complex healthcare situations (Cheraghi et al., 2023).

Ethics education fosters key competencies such as empathy, accountability, ethical decision making, and honest communication. It enhances moral sensitivity and critical thinking, ultimately improving nursing care and ethical practice (Bueno et al., 2023; Shadi et al., 2024).

## Review of the Literature

Research has shown that ethics education in undergraduate nursing programs, particularly in clinical settings or simulations, helps students develop into ethically competent professionals ([Bibr bibr13-23779608261436765][Bibr bibr13-23779608261436765]; Monteverde, 2016; Robinson et al., 2014). Clinical experiences in healthcare settings play a significant role in shaping nurses’ ethical actions and decision making (Sharifikia et al., 2024).

Preparing student nurses for the ethical challenges of clinical practice is vital, and high-quality ethics education should be a fundamental part of nursing training (Munangatire et al., 2023). As an integral part of their education, clinical placements provide nursing students with firsthand experience of ethical challenges in healthcare settings, which may arise either by observing nursing staff or by experiencing directly (Albert et al., 2020; Rabori et al., 2019; Sinclair et al., 2016). The lack of knowledge, insufficient clinical experience, and low confidence can hinder nurses’ ability to navigate morally ambiguous situations, affecting their learning and development (Albert et al., 2020; Bickhoff et al., 2017; Sasso et al., 2016). Therefore, reflecting on these experiences is important for their professional growth and ethical sensitivity (Sinclair et al., 2016). Ethical education encourages critical thinking, enhances self-awareness, and improves clinical practice by helping students take a more objective approach to patient care (Naber & Markley, 2017). Additionally, involves assessing their preparedness to handle situations that may challenge their values and beliefs, as well as their ability to engage in a deliberative process that respects legal regulations and fundamental ethical principles (González-Pérez et al., 2024). Nursing educators must prepare new nurses by providing learning experiences that encourage reflection and the resolution of ethical dilemmas. This preparation requires not only moral knowledge but also moral sensitivity and courage (Escolar-Chua, 2018).

Many challenges nursing students face in the real world of healthcare settings have been highlighted by the literature. Ethical issues such as conflicts with physicians, discrimination based on patients’ socioeconomic status, privacy concerns, and informed consent issues (Font Jiménez et al., 2023). Additionally, students reported multiple ethical concerns, including violations of human and social rights, breaches of confidentiality and privacy, restrictions on patient autonomy, and instances of neglect that were not addressed. Communication issues, such as withholding information from patients and failing to ensure confidentiality, were also observed (Albert et al., 2020; Jasemi et al., 2018).

Nursing students often witness instances of mistreatment by healthcare professionals (HCPs) and the dissemination of misleading or incomplete information (Feeg et al., 2021; Ramos et al., 2015). These experiences underscore the critical need for nursing education to prepare students not only to recognize but also to respond effectively to ethical challenges encountered in clinical practice. While ethics education is often delivered through classroom discussions, such theoretical approaches may be insufficient in preparing students for the complex realities of clinical practice. For this reason, examining students’ real-world clinical experiences is essential for understanding the ethical issues they faced—issues that primarily involve external events and observations rather than solely emotional or subjective reactions. In this study, students were asked to explore the ethical issues they encountered during their clinical training, providing insight into how these situations shape their ethical awareness and development. Although international research has examined the ethical problems nursing students face in various healthcare contexts, evidence in Cyprus remains limited. This gap makes difficult the development of culturally and contextually appropriate educational strategies and support mechanisms.

## Aim of This Study

This study aimed to explore the ethical issues nursing students faced during their four-year clinical training.

## Research Question

*What ethical issues did you face during your clinical practice, based on the “Nursing Ethics and Professional Legislation” module you have attended?*

## Methodology

### Study Design

A qualitative deductive thematic analysis was conducted. This approach is particularly suitable when the aim is to develop themes and interpretations based on a pre-existing theoretical framework, established concepts, or prior literature (Hsieh & Shannon, 2005; Green & Thorogood, 2018), including concepts taught in the Nursing Ethics and Professional Legislation module.

### Setting

The study was conducted within the department of nursing at a university in Cyprus. The program follows the European Directive 2005/36/EC for professional qualifications, ensuring the inclusion of ethics and professional legislation in the nursing curriculum.

### Participants

The study was conducted with fourth year (final year) nursing students. A purposive sample of 57 nursing students were recruited all of whom had completed the “Nursing Ethics and Professional Legislation” module and had accumulated clinical exposure across the four-year curriculum. This enabled participants to reflect retrospectively on ethical issues encountered during earlier placements, using shared ethical vocabulary and the legal/ethical concepts taught in the module. Participants were asked to identify and describe ethical issues they had experienced during clinical practice.

Inclusion criteria required nursing students to:

- Be enrolled in the fourth (final) year of the undergraduate nursing program. Including only final-year nursing students, ensured a homogeneous level of clinical responsibility and ethical competence, as ethical competence is more consolidated in senior nursing students, whose advanced clinical exposure enhances their ability to recognize, articulate, and critically reflect an ethical issue using established theoretical concepts (Font Jiménez et al., 2023).
- Be actively engaged in a clinical placement in hospital settings at the time of the study.
- Successfully completed the module “Nursing Ethics and Professional Legislation,” provided the theoretical framework for the study.
- Have provided written informed consent to participate voluntarily.

Students who were not in clinical placement during the study period or who did not provide consent were excluded.

### Data Collection

Data was collected during the final two lectures of the “Nursing Ethics and Professional Legislation module.” Students submitted their experiences on ethical issues using pseudonyms to maintain confidentiality. The data was sent directly to the main researcher, who verified the use of pseudonyms and proceeded to code. To ensure accuracy in reporting, illustrative experts were translated into English (target language) for publication purposes and subsequently back translated into Greek (source language). Two bilingual team members completed the translation and back translation to ensure data rigor and credibility. Both had strong language proficiency and cultural insight, essential for maintaining the meaning of participants’ observations (Chen & Boore, 2010).

### Data Analysis

To address the research question, a deductive thematic analysis was conducted, following the framework outlined by Braun and Clarke (2006). Braun and Clarke (2006) define thematic analysis as “a method for identifying, analyzing, and reporting patterns (themes) within data,” highlighting its utility in uncovering meaning and structure within complex qualitative datasets. In contrast, qualitative content analysis emphasizes code frequency and involves less interpretative depth (Vaismoradi et al., 2013).

To strengthen analytic rigor, procedures were used to support coding consistency and resolve disagreements within the research team. Two researchers independently coded the dataset following Braun and Clarke's (2006) framework. Coding comparisons were discussed in iterative meetings, referencing the original data until a consensus was reached. When consensus was not reached, a third researcher reviewed the data and contributed to the final decision. These steps informed refinement of the developing codebook and enhanced the credibility and dependability of the analytic process (Braun & Clarke, 2006; Lincoln & Guba, 1985).

The following six steps were.

1. Getting acquainted with the data: Researchers reviewed students’ data repeatedly, noted ideas, and acquired an overall impression.
2. Generating initial codes (deductive coding): Using pre-existing theoretical concepts drawn from the “Nursing Ethics and Professional Legislation” module (e.g., Patients’ Rights, Professional Liability, and Nursing Codes of Ethics), the research team codes data excerpts accordingly. The deductive thematic analysis was guided by the core ethical principles taught in the nursing ethics module, including autonomy, informed consent, equality and justice, confidentiality, privacy, duty of care, professional accountability, and moral integrity. These principles shaped both the coding framework and the interpretation of students’ written responses. The analysis focused on ethical issues related to patient autonomy, informed consent, communication, cultural and religious respect, privacy, and access to medical records—reflecting the module's emphasis on protecting patient rights and promoting dignified, transparent care. Moral integrity informed the identification of moral distress, particularly when students recognized the ethically appropriate action but were unable to act. Principles of professional accountability and the Nursing Codes of Ethics guided the interpretation of communication failures, negligence, and safety lapses.
3. Data grounded under broader codes: Codes were categorized under broader themes, consistent with the deductive framework and the study's exploration of ethical dimensions.
4. Reviewing themes: The research team reviewed the themes and discussed them based on the codes and data, refining and identifying relevant patterns.
5. Defining and naming themes: Themes were clearly defined by the researchers and named accurately, representing the data. An illustrative example of the identification and definition of a subtheme about the violation of privacy and dignity is provided in Table 1.
6. Writing the report: A final report presents the identified themes and insights.

**Table 1.** Defining and Naming Themes.

***Subtheme (Ethical Principle)***	**Code**	**Data Excerpt**	**Summary/Meaning**
	Exposure during care	*“I told the nurse to close the curtains…* *he replied that we don’t have to…”* (SN7)	The patient's body was exposed unnecessarily, a lack of respect for privacy
*Violation of Privacy and Dignity*	No use of curtains between beds	*“Health professionals do not close the curtains separating the two beds…”* (SN16)	The visibility of patients’ bodies to others raises concerns regarding privacy and dignity in clinical environments
	Bed baths without coverage	*“Bed baths and patient exposure without covering the bodies…”* (SN21)	Patient dignity compromised; inadequate measures to protect modesty

#### Ethical Considerations

Ethical approval for the study was obtained from the Department of Nursing. This study was conducted in full accordance with the ethical principles outlined in the Declaration of Helsinki (World Medical Association, 2013), which emphasizes respect for human dignity, the necessity of informed consent, and the protection of participants’ rights and wellbeing. All participants were provided with detailed information regarding the objectives of the study, the voluntary nature of their involvement, and their right to withdraw from the research at any point without any adverse consequences. Written informed consent was obtained from each participant before data collection. To ensure confidentiality and anonymity, no personal identifiable information was collected. Participants were explicitly instructed to omit any references to specific hospitals, departments, or individuals when describing ethical incidents. All data were securely stored and made accessible exclusively to the research team. By adhering to the ethical standards, the study ensured the protection of participants’ dignity, autonomy, and rights throughout the research process (Beauchamp & Childress, 2013; World Medical Association, 2013). The data were stored in a locked cabinet in the researcher's office, with access limited to the researcher.

#### Rigor

To ensure the transferability of this deductive qualitative study, various strategies were implemented to strengthen credibility, dependability, confirmability, and transferability, in accordance with the evaluative criteria established by Lincoln and Guba (1985). The techniques and procedures employed to ensure trustworthiness are detailed in Table 2. These practices ensured that the results authentically represented participants’ perspectives (Hanson et al., 2019).

**Table 2.** Trustworthiness Criteria According to Lincoln and Guba (1985).

***Criteria***	**Techniques Performed and Application Procedures**
*Credibility*	Predefined themes remained grounded in student narratives for authentic representation. Credibility, according to Lincoln and Guba, was ensured through data immersion, peer debriefing, member checking, and independent review by a senior researcher
*Transferability*	Transferability was ensured through rich student descriptions and verbatim quotes, allowing readers to assess applicability to other contexts
*Dependability*	Dependability was ensured via a comprehensive audit trail of research decisions, supporting evaluation of the study's consistency and repeatability
*Confirmability*	Confirmability was ensured through a detailed audit trail, researcher reflexivity, and grounding interpretations in participants’ statements. Verbatim excerpts further enhanced transparency and rigor, ensuring the findings authentically reflect participants’ perspectives (Hanson et al., 2019)
	

## Results

### Sample Characteristics

Of 57 eligible fourth-year nursing students, 55 participated (response rate: 96.5%). The sample included 49 females (89.1%) and 6 males (10.9%), reflecting the gender distribution of the nursing program. Participants’ ages ranged from 22 to 26 years.

### Study Themes

The analysis of the written data revealed three key themes, and nine subthemes (Tables 3 and 4). The three main themes identified were (1) Violation of Patients’ Rights, (2) Moral Distress, and (3) Professional Liability and Nursing Codes of Ethics. Reporting quality (Table 5) was evaluated according to the Standards for Reporting Qualitative Research (SRQR), a 21-item checklist (O’Brien et al., 2014).

**Table 3.** Codes That Emerged in the Category of Patients’ Rights.

**Autonomous Decision Making**	**Instances Where Patients Were Not Allowed to Make Their Own Decisions**
**Informed** c**onsent and** i**nsufficient** i**nformation**	Cases where permission was not properly obtained from patients or where consent forms were inadequately handled. Additionally, there were situations where patients were not given enough information about their condition
**Equality and** r**eligious** f**reedom**	Violations related to patients’ rights to equality and freedom of religious beliefs
**Access to** m**edical** r**ecords**	Instances where patients’ access to their medical records was restricted
**Privacy** v**iolations**	Breaches of patient privacy

**Table 4.** Themes, Subthemes, and Codes Emerge From the Data.

**Main Themes**	**Sub**t**hemes**	**Codes**
**Patients’** R**ights**	Autonomous decision making	Restricted autonomy in decision making regarding access to hospitals Limited autonomy in treatment-related decision making Compromised autonomy in end-of-life decisions
	Informed consent and insufficient information	Failure to disclose truthful and complete information Inadequate communication with patient/family Insufficient details about the patients’ condition Failure to provide appropriate information according to the level of knowledge and understanding of the patient Violation of obtaining signed consent
	Equality and religious freedom	Racist behavior Failure to provide information based on the ethnicity of the patient Violation of the practice of religious beliefs
	Access to medical records	Violation of patients’ right to access medical records
	Privacy	Exposure of the patient's body
**Moral Distress**	Pressure among health professionals	Anxiety, pressure, and stress Moral awareness and the ideal course of action
**Professional Liability and Nursing Codes of Ethics**	Lack of proper nurse–patient communication	Duty of care/accountability Negligence

**Table 5.** Standards for Reporting Qualitative Research*.

Title and Abstract	Page/Line No(s).
*Title*: Concise description of the nature and topic of the study identifying the study as qualitative or indicating the approach (e.g., ethnography and grounded theory) or data collection methods (e.g., interview, focus group) is recommended	Title, page 1
*Abstract*: Summary of key elements of the study using the abstract format of the intended publication; typically includes background, purpose, methods, results, and conclusions	Abstract, page 1
*Introduction*	
*Problem formulation*: Description and significance of the problem/phenomenon studied; review of relevant theory and empirical work; problem statement	Introduction, pages 2–3
*Purpose or research question*: Purpose of the study and specific objectives or questions	Purpose of the study, page 5
*Methods*	
*Qualitative approach and research paradigm*: Qualitative approach (e.g., ethnography, grounded theory, case study, phenomenology, narrative research) and guiding theory if appropriate; identifying the research paradigm (e.g., postpositivist, constructivist/interpretivist) is also recommended; rationale**	Methods, settings, pages 5–6
*Researcher characteristics and reflexivity*: Researchers’ characteristics that may influence the research, including personal attributes, qualifications/experience, relationship with participants, assumptions, and/or presuppositions; potential or actual interaction between researchers’ characteristics and the research questions, approach, methods, results, and/or transferability	Ethical considerations, page 8
*Context*: Setting/site and salient contextual factors; rationale**	Study setting, sample of the study, page 5
*Sampling strategy*: How and why research participants, documents, or events were selected; criteria for deciding when no further sampling was necessary (e.g., sampling saturation); rationale**	Study setting, sample of the study, pages 5–6
*Ethical issues pertaining to human subjects*: Documentation of approval by an appropriate ethics review board and participant consent, or explanation for lack thereof; other confidentiality and data security issues	Ethical considerations, page 8
*Data collection methods*: Types of data collected; details of data collection procedures including (as appropriate) start and stop dates of data collection and analysis, iterative process, triangulation of sources/methods, and modification of procedures in response to evolving study findings; rationale**	Data collection, pages 6–7
*Data collection instruments and technologies*: Description of instruments (e.g., interview guides, questionnaires) and devices (e.g., audio recorders) used for data collection; if/how the instrument(s) changed over the course of the study	Described in data analysis, pages 6–8
*Units of study*: Number and relevant characteristics of participants, documents, or events included in the study; level of participation (could be reported in results)	Sample, pages 5–6
*Data processing*: Methods for processing data prior to and during analysis, including transcription, data entry, data management and security, verification of data integrity, data coding, and anonymization/de-identification of excerpts	Data analysis, pages 6–7
*Data analysis* Process by which inferences, themes, etc., were identified and developed, including the researchers involved in data analysis; usually references a specific paradigm or approach; rationale**	Data analysis, pages 6–8
*Techniques to enhance trustworthiness*: Techniques to enhance trustworthiness and credibility of data analysis (e.g., member checking, audit trail, triangulation); rationale**	Rigor, pages 8–9 and Table 2
	
*Results/findings*	
*Synthesis and interpretation*: Main findings (e.g., interpretations, inferences, themes); might include development of a theory or model, or integration with prior research or theory	Results, pages 9–14
*Links to empirical data*: Evidence (e.g., quotes, field notes, text excerpts, photographs) to substantiate analytic findings	N/A
*Discussion*	
*Integration with prior work, implications, transferability, and contribution(s) to the field*: Short summary of main findings; explanation of how findings and conclusions connect to, support, elaborate on, or challenge conclusions of earlier scholarship; discussion of scope of application/generalizability; identification of unique contribution(s) to scholarship in a discipline or field	Discussion, pages 14–19
*Limitations*: Trustworthiness and limitations of findings	Page 18

*, ** Thematic analysis.

**Reference:** O’Brien et al. (2014).

#### Theme 1: Violation of Patients’ Rights

Nursing students have observed actions and omissions that compromised patients’ and relatives’ autonomy across multiple aspects of care. Codes that emerged under autonomous decision making included: (1) restricted autonomy in decisions regarding hospital access, (2) limited autonomy in treatment-related decisions, and (3) compromised autonomy in end-of-life decisions. For example, a family attempted to transfer their hospitalized relative to a private clinic to pursue further diagnostic testing and explore alternative treatment options. However, their request was denied by both the attending physician and the nursing staff, constraining their freedom to choose another healthcare facility.

1. Restricted autonomy in decision making concerning hospital access.
  “…relatives were protesting and wanted to leave the hospital and go to a private clinic. The doctor who was treating her and some nurses told them that they could not leave the hospital.” SN17
  This denial represents a clear breach of the patient's and family's right to make informed decisions regarding their care, highlighting the limited agency they had in determining treatment and continuity of care.
2. Limited autonomy in treatment-related decision making
3. student nurse (SN4) recalled a situation where a patient, feeling uninformed and distressed, repeatedly asked: “*Why are you standing like statues and not telling me what I have*?” The student described making every effort to support the patient despite being constrained by limited authority within the clinical environment. This example illustrates the ethical tension nursing students experience when communication with patients is restricted, potentially undermining patient autonomy and informed consent.
4. Compromised autonomy in end-of-life decisions.
5. ne student described an emotionally distressing situation in palliative care. After a procedure, the patient expressed despair, staying: “*I prefer to die than be in pain. I wish you would put something in me to die*.” Such expressions reveal how unrelieved pain, emotional distress, and psychological burden can diminish a patient's ability to make autonomous decisions. This highlights the ethical and clinical need for comprehensive pain management, empathetic communication, and interdisciplinary collaboration involving physicians, palliative care teams, and psychological support.

#### Informed Consent and Insufficient Information

The lack of informed consent emerged as a major ethical concern. Informed consent is central to nursing ethics and legal practice, ensuring that patients are adequately informed about, and voluntarily agree to, their care. Within this subtheme, five distinct codes emerged: (1) failure to disclose truthful and complete information, (2) inadequate communication with patients and families, (3) insufficient details about the patient's condition, (4) failure to adapt information to the patient's level of knowledge and understanding, and (5) violation of procedures for obtaining consent.

1. Failure to disclose truthful and complete information
  “I also noticed the lack of information from health professionals regarding the procedures they perform and the absence of information regarding their health condition, resulting in ignorance of patients and increased anxiety about the outcome of their illness.” SN26

This statement reflects a major gap in patient-centered communication. The absence of honest, and complete information undermines informed decision making, heightens anxiety, and weakens trust in care relationships.

2. Inadequate communication with patients and families
  “Patients and their families had little or no information regarding their health condition, thus leading to constant conflicts with health professionals.” SN50

Students consistently emphasized the ethical importance of transparent and compassionate communication to preserve trust and ensure informed consent.

3. Insufficient details about the patient's condition
  “Health professionals gave incomplete information on the treatment methods they used on patients.” SN21

The issue of inadequate communication regarding patients’ health conditions emerged as a recurring concern among student nurses. Several students observed that healthcare professionals often provided incomplete or superficial information about treatment methods and clinical procedures. This lack of transparency extended beyond the patients themselves, affecting their families and often resulting in confusion and tension. As evidenced by SN50, limited information led to frequent conflicts between families and healthcare providers.

4. Failure to tailor information to the patient's level of understanding
  “We had to go to an elderly woman with dementia and ask her to sign the consent form for her transfer to the operating room. […] However, the supervisor asked us to tell her to sign without any information on what would happen.” SN51

A critical ethical concern identified by student nurses relates to the failure of healthcare professionals to tailor information according to the patient's cognitive capacity and level of understanding. As illustrated in SN51, a student described being instructed to obtain informed consent from an elderly woman with dementia before surgery. Despite the patient's evident cognitive impairment and inability to comprehend the situation, the student was directed by a supervisor to secure a signature without offering any explanation about the procedure.

5. Violation of obtaining signed consent
  Further concerns were raised regarding the violation of both ethical and legal principles of informed consent, particularly the absence of adequate explanation before obtaining a patient's signature.

#### Equality and Religious Freedom

Students reported multiple cases where patients’ rights to equal treatment and religious freedom were disregarded. These violations often stemmed from discriminatory attitudes or communication failures by HCPs. The main codes identified under the theme “Equality and Religious Freedom” include:

1. Racist behavior
  “Due to her origin being from a third-world country, the behavior of the health professional was neither equal nor decent.” SN1

This incident reflects a clear breach of the principles of equitable care, which require that all patients—regardless of their ethnic, cultural, or national background, receive the same level of respect, compassion, and professional treatment. Such discriminatory conduct not only violates the patient's right to equality but can also inhibit the expression of their religious or cultural identity within the healthcare environment.

2. Failure to provide information based on patients’ ethnicity
  “This patient was of foreign origin… for 3–4 days that he was hospitalized, it was not possible to inform him about any issues related to his health.” SN11

This illustrates the absence of culturally and linguistically appropriate communication, which limits patient understanding and informed decision making.

3. Violation of religious practices
  “She will eat whatever we have.” SN8

Student nurses reported several cases in which patients’ religious beliefs and cultural practices were disregarded by HCPs. Such dismissive responses toward patients’ dietary or religious needs reflect cultural insensitivity and disregard for respect in care.

#### Access to Medical Records

Students noted that patients were sometimes denied access to their medical records, raising serious concerns about transparency and respect for patient rights: “*Ward staff, including nurses, refused to give records to the patient*” (SN12).

#### Privacy

Students frequently observed breaches of privacy that compromised patient dignity: “*The nurse dismissed my request to close the curtains during wound care, arguing it was unnecessary since the procedure was brief*” (SN7).

Such behaviors undermine ethical principles of respect and confidentiality.

#### Theme 2: Moral Distress

Although not the predominant theme, students described experiencing moral distress and emotional strain, especially in palliative care and interactions with patients’ relatives. However, the theme of “Moral Distress” emerged through two key codes: (1) anxiety, pressure, and stress, and (2) moral awareness and the ideal course of action.

#### Pressure Among Health Professionals

1. Anxiety, pressure, and stress
  “I began to wonder… Why this palliative care… It caused me anxiety, pressure, and stress… I had moral distress.” SN41

These reflections illustrate the emotional burden of ethically complex clinical situations and the need for structured psychological and ethical support during the training of the students.

2. Moral awareness and the ideal course of action
  “I appreciate you, but I am psychologically tired of the whole situation and of the whims of the relatives.” SN42

These reflections highlight the importance of promoting supportive environments and implementing strategies to help HCPs manage moral distress effectively, thereby protecting the wellbeing of both staff and patients. They also emphasized the need to cultivate emotional resilience and provide structured opportunities for ethical reflection within nursing education.

#### Theme 3: Professional Liability and Nursing Codes of Ethics

Nursing students identified several breaches of professional and ethical standards outlined in the Cypriot Codes of Nursing Ethics and the 2012 Standard Practice Regulations. These included poor nurse–patient communication, negligence, discriminatory behavior, and lack of accountability.

1. Duty of Care /Accountability
  Students reported several cases in indicating lapses in duty of care and professional accountability, particularly in relation to patient safety and responsiveness. These included cases where nurses ignored patients repeated calls for help (SN21) and failed to implement basic safety measures, such as raising bed rails or ensuring access to the call bell (SN53). Such behaviors reflect negligence and disregard for both ethical standards and patient welfare.
2. Negligence
  “The nurse left the room after returning the patient to his bed without placing the protective rails and doorbell near the patient.” SN53

These observations underscore the need for continuous professional training and monitoring to ensure ethical compliance and patient safety.

## Discussion

The findings of this qualitative study reveal critical ethical and professional challenges in nursing practice, particularly concerning patients’ rights, moral distress among nurses, and adherence to nursing codes of ethics. Three major themes emerged from the student nurses’ observations: Violation of Patients’ Rights, Moral Distress and Pressure among Health Professionals, and Professional Liability and Nursing Codes of Ethics. Collectively, these themes highlight the complex ethical landscape that nursing students encounter during clinical placements. Comparable findings have been reported internationally. For example, research conducted among Malawian nursing students identified similar ethical concerns related to informed consent, refusal of treatment, privacy and confidentiality, respect and dignity, equality and justice, and experiences of moral distress (Solum et al., 2012).

Numerous studies have emphasized the inadequate protection of patients’ rights, a recurring theme in research involving nursing students (Sinclair et al., 2016; Solum et al., 2012; Tsuruwaka, 2017). This persistent issue represents a major ethical concern within clinical practice, and poses particular challenges for student nurses who are still developing their understanding of ethical principles and patient-centered care. The present findings reveal recurrent breaches of patient autonomy, with several cases illustrating the denial of patients’ or relatives’ ability to make informed decisions about their care. Examples include the refusal to allow a patient's relative to arrange a hospital transfer and the unilateral decision making regarding the placement of an elderly patient in long-term care. Such instances reflect a disregard for individual choice and self-determination, core tenets of ethical nursing practice. These results are consistent with previous studies that have similarly documented the insufficient protection of patients’ rights as observed by nursing students (Ramos et al., 2015; Sinclair et al., 2016; Solum et al., 2012).

The findings also revealed ethical challenges in end-of-life care, raising ethical questions about the adequacy of palliative services and the extent to which patients’ wishes are respected in such circumstances. These findings echo broader debated in the literature regarding conflicts that often arise between healthcare providers and patients in end-of-life decision making (Jahn Kassim & Alias, 2016). In the Cypriot context, such tensions may be influenced by the legal prohibition of euthanasia (Televantos et al., 2013). Consequently, nursing professionals should prioritize clear empathetic communication with patients and families, ensuring that they understand that euthanasia is not legally permissible, while simultaneously advocating for the strengthening of palliative care to provide compassionate and ethically sound end-of-life support.

A recurring issue in this study was the lack of informed consent and failure to provide adequate information to patients. Previous studies have emphasized the importance of transparency and truth-telling in relation to patients’ health status and treatment progress (Cevik Durmaz, 2022). The absence of clear explanations or the performance of unconsented procedures represents a serious ethical violation and a disregard for patient autonomy. These shortcomings were often compounded by poor communication regarding patients’ medical conditions, which in turn led to heightened anxiety, mistrust, and conflicts between patients and HCPs (Cevik Durmaz, 2022; Rabori et al., 2019; Ramos et al., 2015).

Instances of discrimination based on nationality or religion and inadequate accommodation of patients’ religious practices were also observed. Such violations not only compromise the dignity and respect owed to patients but also undermine the fundamental principles of equality and fairness in healthcare (Hansen et al., 2024). Research has shown that HCPs exhibiting ethnocentric attitudes may overlook the cultural, religious, or ethnic backgrounds of patients, contributing to poor health outcomes (Kaya et al., 2021). Moreover, a lack of cultural competence training among nurses and other healthcare staff further limits their ability to provide equitable care (Jirwe et al., 2010). Despite ongoing efforts to promote inclusivity and patient advocacy, these findings suggest that advocacy is not yet practiced effectively, resulting in limited protection and promotion of patients’ rights, needs, and interests.

The denial of access to medical records and breaches of privacy reported by students raise serious ethical concerns regarding transparency and respect for patient confidentiality. Instances in which patients and their families were denied essential information, or where privacy was neglected during care, point to systemic shortcomings in upholding patients’ fundamental rights. Such practices not only violate ethical and legal standards but also weakness trust in healthcare institutions. These findings are consistent with previous research highlighting persistent gaps in ensuring confidentiality and transparency in patient care (Hosseini et al., 2024; Solum et al., 2012).

The study also revealed that nursing students frequently observed moral distress among their peers, particularly in ethically challenging situations such as palliative care and interactions with difficult relatives. This distress was often associated with ethical dilemmas and perceived violations of patient rights. Previous research has highlighted the detrimental effects of moral distress on both nurses and nursing students in clinical settings (Krautscheid et al., 2017; Solum et al., 2012). Such distress typically arises when individuals recognize the ethically appropriate course of action but feel constrained from acting accordingly. Moreover, moral distress among student nurses has been linked to communication barriers with patients, feelings of powerlessness in the presence of physicians, and the provision of misleading information to patients regarding their treatment (Sasso et al., 2016).

Several factors contributing to moral distress among nursing students are associated with resource shortages and their improper allocation. These factors include the hierarchical dynamics between doctors and nurses, the profound sense of isolation often experienced by trainees, and the perception of limited autonomy among trainers within the clinical training environment (Sasso et al., 2016; Wojtowicz et al., 2014).

Compared to registered nurses, nursing students possess less experience, skill, and confidence in managing ethically challenging situations, particularly within clinical settings ([Bibr bibr3-23779608261436765][Bibr bibr3-23779608261436765]). Our findings suggest areas of tension with the principles articulated in the International Code of Nursing Ethics (ICN, 2021). However, professional codes alone may not provide sufficient theoretical leverage to interpret how students reason through ethical complexity. To deepen the analysis, the findings can be situated within established ethical theories, highlighting both explanatory value and limitations. Principlism (Beauchamp & Childress, 2013) helps explain conflicts among ethical principles in practice (e.g., autonomy, beneficence, nonmaleficence, and justice) but does not fully capture the relational and emotional dimensions of participants’ experiences. Care ethics (Gilligan, 1993) provides a complementary perspective by emphasizing relational responsibility, vulnerability and context-sensitive moral judgment. Additionally, virtue ethics (Monteverde, 2016; Hansen et al., 2024) conceptualizes ethical competence as a developmental and practice-based aligning with students’ descriptions of learning ethical action over time, while our findings also point to structural and organizational constraints that can limit individual moral agency. Taking together, these perspectives frame ethical competence as a dynamic, context-dependent capacity rather than mere adherence to rules (Hansen et al., 2024; Santos et al., 2025; Zhu et al., 2025).

Violations reported by nursing students—such as discriminatory treatment, verbal abuse, and neglect—highlight a profound gap between the realities of clinical practice and the ethical principles set forth in professional codes (Albert et al., 2020; Rabori et al., 2019). A study conducted in Ghana similarly revealed that nurses’ responses to ethical issues often diverged from the standards established by the International Council of Nurses (Donkor & Andrews, 2011). Instances of negligence, discriminatory behavior and physical mistreatment signify a serious breach of professional conduct and patient care. The occurrence of unprofessional behavior among nurses has been consistently documented in previous research (Sinclair et al., 2016; Solum et al., 2012). Likewise, nursing students in Malawi reported frequent ethical violations including ignoring patient needs, shouting at patients, and taking “shortcuts” in care delivery—practices that mirror the findings of the present study (Solum et al., 2012).

### Strengths and Limitations

This study is the first of its kind in Cyprus to document ethical issues as identified by nursing students within hospital settings. The findings provide valuable insights into students’ perceptions of ethical challenges, offering a nuanced portrayal of the clinical environment through their lived experiences. These perspectives can inform the development of educational and institutional interventions aimed at fostering ethical awareness and minimizing ethical conflicts in nursing practice. Furthermore, the study enhances understanding of the ethical dimensions of nursing education within the Cypriot context, establishing a foundation for future research involving a larger and more diverse cohort of students.

However, several limitations must be acknowledged. The qualitative design and the focus on fourth year (final-year) nursing students limit the transferability of the findings to other educational levels and contexts and educational levels. In addition, conducting the study within a single university and restricting the sample to final-year nursing students, limits the representativeness of the sample and may under-represent experiences from earlier-year students. From a methodological perspective, the absence of triangulation and member checking reduces the study's credibility and dependability, as outlined by Lincoln and Guba (1985). Incorporating multiple data sources, researcher reflexivity, and participant validation could have enhanced the confirmability and sources, researcher reflexivity and participant validation could have enhanced the confirmability and robustness of the results. Future research should aim to include students from various institutions and employ diverse qualitative approaches to strengthen the trustworthiness of findings and provide a more comprehensive understanding of ethical challenges within Cypriot healthcare settings.

### Implications for Practice

The findings of this study have several implications for nursing practice and education in Cyprus. First, they emphasize the need to integrate ethics into clinical training through reflective discussions, supervision, and simulation exercises addressing real ethical dilemmas. Second, healthcare institutions should strengthen patient rights and communication protocols to promote informed consent, privacy, and equality. Third, developing support systems for nursing students who experience moral distress, such as mentoring and ethics reflection groups, can enhance emotional resilience and ethical competence. Finally, collaboration between universities and healthcare institutions is very important to establish national standards for ethical nursing practice and to promote a culture of accountability, empathy, and respect within Cypriot healthcare settings.

## Conclusion

The study demonstrated that nursing students encounter ethical issues comparable to those identified in previous research. Addressing these challenges is essential for enhancing patient care and reinforcing the ethical principles that underpin nursing practice. The findings highlighted two key concerns: first, students initially developed an understanding of ethical challenges in theory and subsequently recognized these dilemmas during clinical practice. Second, they identified the persistent presence of ethical issues within hospital settings. Despite established legislation protecting patients’ rights, numerous violations continue to occur underscoring the need for stronger ethical education, institutional accountability, and policy enforcement within Cypriot healthcare environments. Furthermore, both academic and clinical settings should provide emotional and professional support to help students manage moral distress and build resilience as future healthcare professionals.
